# Coxsackievirus A10 atomic structure facilitating the discovery of a broad-spectrum inhibitor against human enteroviruses

**DOI:** 10.1038/s41421-018-0073-7

**Published:** 2019-01-15

**Authors:** Jinhuan Chen, Xiaohua Ye, Xue-Yang Zhang, Zhengdan Zhu, Xiang Zhang, Zhijian Xu, Zhanyu Ding, Gang Zou, Qingwei Liu, Liangliang Kong, Wen Jiang, Weiliang Zhu, Yao Cong, Zhong Huang

**Affiliations:** 1 National Center for Protein Science Shanghai, State Key Laboratory of Molecular Biology, CAS Center for Excellence in Molecular Cell Science, Shanghai Institute of Biochemistry and Cell Biology, Chinese Academy of Sciences, University of Chinese Academy of Sciences, Shanghai, China; 2 CAS Key Laboratory of Molecular Virology & Immunology, Institut Pasteur of Shanghai, Chinese Academy of Sciences, University of Chinese Academy of Sciences, Shanghai, China; 3 CAS Key Laboratory of Receptor Research, Drug Discovery and Design Center, Shanghai Institute of Materia Medica, Chinese Academy of Sciences, University of Chinese Academy of Sciences, Shanghai, China; 4 Shanghai Science Research Center, Chinese Academy of Sciences, Shanghai, China; 5 Markey Center for Structural Biology, Department of Biological Sciences, Purdue University, West Lafayette, IN USA

**Keywords:** Cryoelectron microscopy, Structural biology

## Abstract

Coxsackievirus A10 (CV-A10) belongs to the *Enterovirus* species A and is a causative agent of hand, foot, and mouth disease. Here we present cryo-EM structures of CV-A10 mature virion and native empty particle (NEP) at 2.84 and 3.12 Å, respectively. Our CV-A10 mature virion structure reveals a density corresponding to a lipidic pocket factor of 18 carbon atoms in the hydrophobic pocket formed within viral protein 1. By structure-guided high-throughput drug screening and subsequent verification in cell-based infection-inhibition assays, we identified four compounds that inhibited CV-A10 infection in vitro. These compounds represent a new class of anti-enteroviral drug leads. Notably, one of the compounds, ICA135, also exerted broad-spectrum inhibitory effects on a number of representative viruses from all four species (A–D) of human enteroviruses. Our findings should facilitate the development of broadly effective drugs and vaccines for enterovirus infections.

## Introduction

Coxsackievirus A10 (CV-A10) belongs to the *enterovirus* genus in the *Picornaviridae* family^1^. The virus is considered one of the major causative agents of hand, foot, and mouth disease (HFMD), which is highly infectious and affects millions of young children annually. CV-A10 infection has been linked to large epidemics of HFMD in different countries including Japan^2^, France^3^, and China^4,5^. Besides mild symptoms such as herpangina and pharyngitis, severe complications or even fatality also occur with CV-A10 infection^4,6,7^. CV-A10 often co-circulates with other HFMD-causing enteroviruses, including enterovirus 71 (EV-A71), coxsackievirus A16 (CV-A16), and/or coxsackievirus A6 (CV-A6)^3–5,8^, therefore complicating the control and prevention of HFMD on the whole. Unfortunately, neither prophylactic vaccine nor therapeutic drug is currently available for treating CV-A10 infection.

Human enteroviruses can be categorized into four species, including A–D (http://www.picornaviridae.com/enterovirus/enterovirus.htm). Human enteroviruses from cell cultures often exist in two forms: one is infectious mature virion (also termed “F-particle”), which contains viral RNA genome, and the other is non-infectious native empty particle (NEP) (also termed “E-particle”) without viral RNA genome^9,10^. High-resolution structural information is available for a number of human enteroviruses, such as EV-A71^11,12^, CV-A16^13,14^, coxsackievirus B3 (CV-B3^15^), CV-A6^16,17^ and poliovirus^18^. In general, enteroviral capsids are composed of 60 copies of biological protomers that are arranged in a pseudo-*T* = *3* icosahedral symmetry. Each protomer consists of four capsid proteins, including VP1, VP2, VP3, and VP4. In mature virions, VP4 together with the N-terminus of VP1 decorate the internal surface of the capsid shell^9,10^. On the outer surface of enteroviral capsids, there are three-fold propeller-like protrusions, star-shaped five-fold plateaus (called “mesa”), and depressions (called “canyon”) surrounding each plateau. The “canyon” region often serves as the site where host receptors insert or bind^19^. Underneath the “canyon” floor, there is a hydrophobic pocket, which is frequently occupied by a host-derived fatty acid-like “pocket factor”^9,10,19^. Expulsion of the “pocket factor” upon virus binding to its receptor is a prelude to a series of uncoating events, leading to release of viral genome into the cytosol. Pocket factors of different enteroviruses may vary in length and orientation^9,10,19^. Efforts have been made to screen and optimize antiviral drugs that strongly bind into the hydrophobic pocket, replace the natural pocket factor and therefore inactivate the virion^20,21^. One of these pocket-binding compounds, pleconaril, has been evaluated in clinical trials and shown promise in treating picornavirus infections^22^. In general, the screening processes relied heavily on testing individual compounds by in vitro infection-inhibition assay and were therefore labor intensive, time consuming, and inefficient.

We and other groups have previously shown that cell culture-derived CV-A10 displays a ~30 nm spherical particle morphology^23,24^. However, high-resolution structure of CV-A10 virion remains unavailable until now. In the present study, we determined the structures of CV-A10 mature virion and NEP at 2.84 and 3.12 Å, respectively, by cryo-EM single-particle analysis. Our structures reveal atomic resolution details of the hydrophobic pocket and the pocket factor of CV-A10, allowing us to perform virtual screening of pocket-binding inhibitors from about 4 million small-molecule compounds by in silico docking. Four of the 258 high-ranking compounds selected from the virtual screening were found to be able to inhibit CV-A10 infection in vitro. Significantly, one of these compounds, designated ICA135, also exhibits inhibitory effects on a panel of viruses from all four (A, B, C, and D) human enterovirus species, thus representing a promising lead compound for pan-human enterovirus inhibitor drug development.

## Results

### Overall structures of the CV-A10 mature virion and NEP

**Fig. 1:**
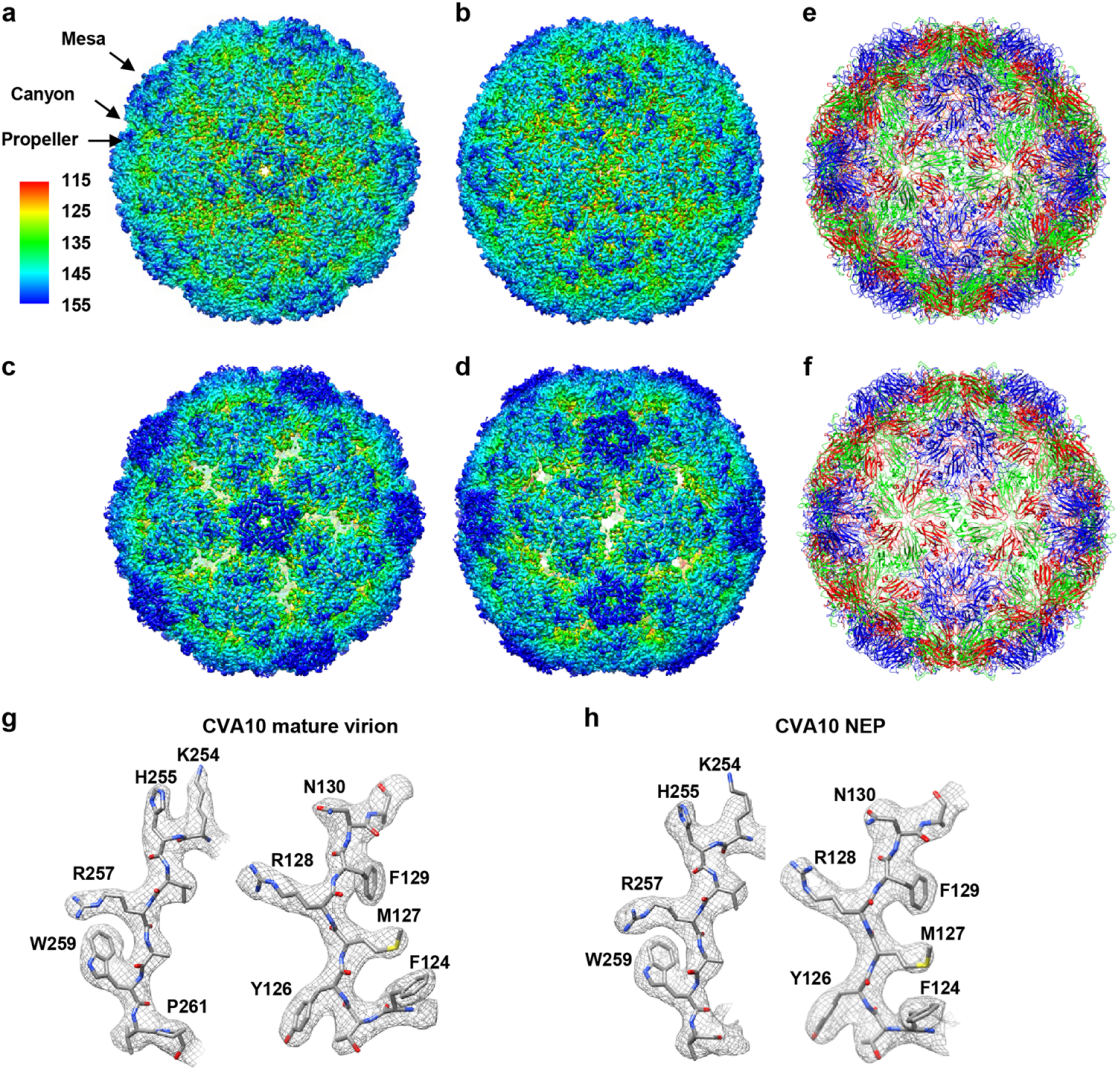
Atomic-resolution cryo-EM structures of CV-A10 particles. **a**, **b** Cryo-EM maps of CV-A10 mature virion viewed along the icosahedral five-fold (**a**) and two-fold (**b**) axis, respectively. The color bar labels the corresponding radius from the center of the sphere (unit in Å). The same color scheme was followed throughout. **c**, **d** Cryo-EM maps of CV-A10 NEP viewed along the icosahedral five-fold (**c**) and two-fold (**d**) axis, respectively. **e**, **f** Atomic models of CV-A10 mature virion (**e**) and NEP (**f**) viewed along the icosahedral two-fold axis, respectively. The models of capsid proteins VP1, VP2, VP3, and VP4 were colored in blue, green, red, and yellow, respectively. The same color scheme was followed throughout, unless otherwise indicated. **g**, **h** Atomic-resolution structural features of CV-A10 mature virion (**g**) and NEP (**h**), respectively, with the segmented density (mesh) in gray and the corresponding atomic model (sticks) in color. The well-resolved densities for almost all the side chains demonstrate the high resolution of the cryo-EM map

Cryo-EM images of purified CV-A10 samples revealed the presence of two types of particles, including filled particles representing the mature virion, and NEP (Supplementary Fig. S1a). A total of 21,783 filled particles and 43,768 NEP particles selected from 2303 images were subjected to 3D reconstruction using jspr software package^25^. The nominal resolutions of the mature virion and NEP density maps are 2.84 and 3.12 Å, respectively (Fig. 1a–d, Supplementary Fig. S1b and Supplementary Table S1). The local resolution of mature virion varies between 2.6 Å (the antiparallel β-barrel core) and 3.3 Å (several highly dynamic surface loops, such as VP1 GH-loop and VP2 EF-loop), while that for NEP is between 2.8 and 4.0 Å evaluated using ResMap^26^ (Supplementary Fig. S1c, d). Our cryo-EM maps clearly reveal the side chain densities of most amino acids, allowing us to build atomic models for each map, with the model and map matching very well (Fig. 1e–h).

The cryo-EM map of mature virion displays surface features typical for enteroviruses, including a “mesa” at five-fold symmetry axis, a “canyon” around the mesa, and a three blade “propeller” at three-fold symmetry axis (Fig. 1a, b, e). In the mature virion, most of the surface exposed hydrophilic loops, including the BC, EF, and GH loops of VP1 and the EF loop of VP2 are well resolved, whereas the densities for a few residues (including residues 1, 10–17, and 297 of VP1, residues 1–10 of VP2, and residues 1–27 and 69 of VP4) are missing. In the NEP map, some surface-exposed loops, such as the GH loops of VP1 and VP3, are unresolved; additionally, residues 1–96, 112–123, and 321–324 of VP0, 1–72 and 297 of VP1, and 176–182 of VP3 are also missing in this map.

### Structural comparison between mature virion and NEP

**Fig. 2:**
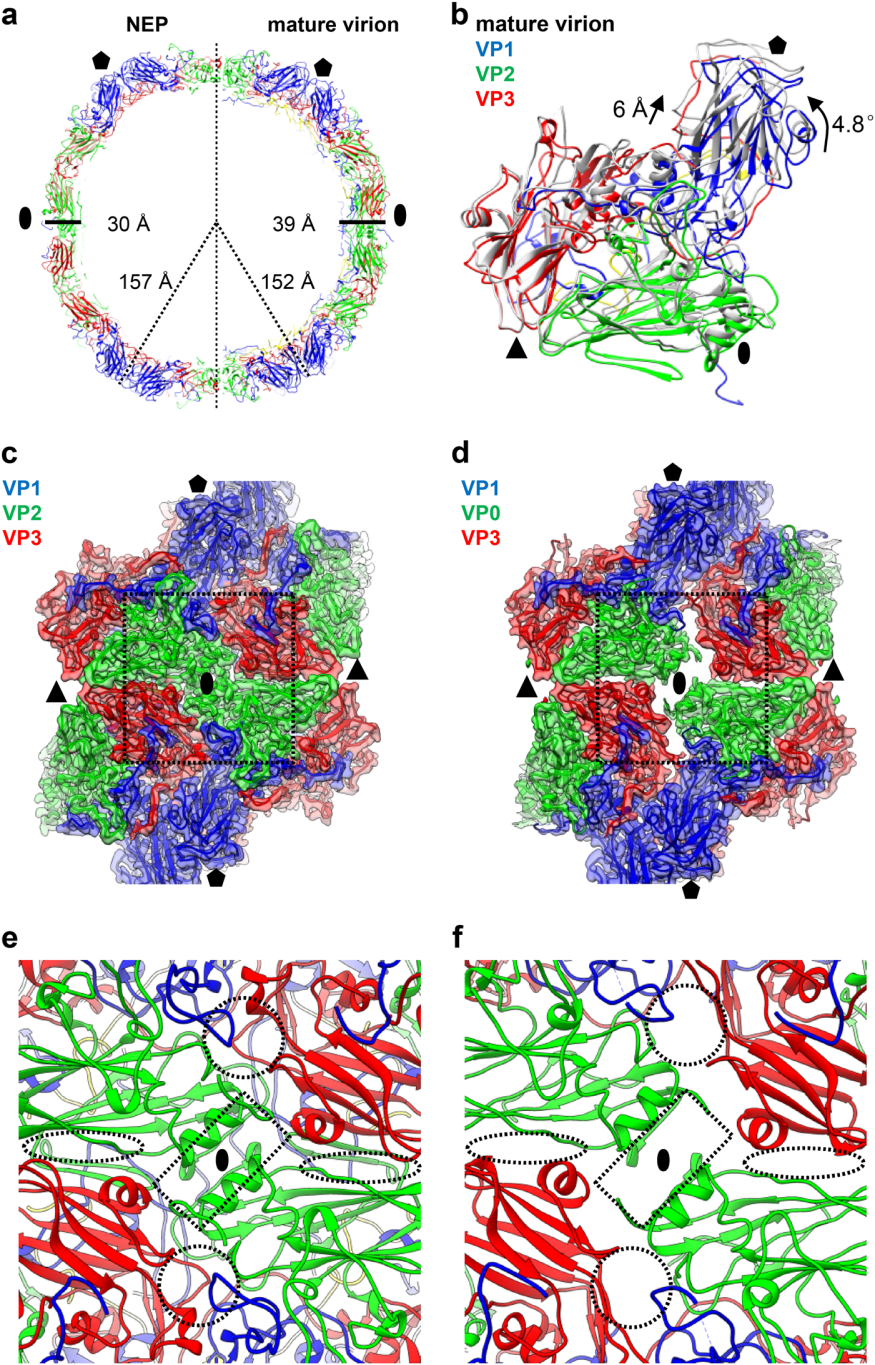
Capsid structural comparison between CV-A10 mature virion and NEP. **a** Two half sections of a 20 Å-thick central slab through the atomic models of CV-A10 NEP particle (left) and mature virion (right). Black oval and pentagon represent the two-fold and five-fold axes, respectively. The capsid radiuses and thickness for the two types of particles are also labeled. **b** One protomeric unit of CV-A10 NEP (in gray) was aligned with that of mature virion (in color). The rotation and translation from mature virion to NEP were also labeled. Black oval, triangle, and pentagon represent the two-fold, three-fold, and five-fold axes, respectively. **c**, **d** Structural configurations of four adjacent protomers around the two-fold axis for CV-A10 mature virion (**c**) and NEP (**d**), respectively. The major differences between them were indicated by dashed rectangle. **e**, **f** Zoom-in view of the icosahedral two-fold region of CV-A10 mature virion (**e**) and NEP (**f**). Dashed rectangle, circle, and oval indicate the locations of two-fold channel, a second channel nearby the quasi-three-fold axis, and another small ditch formed at the VP2/VP3 interface between adjacent protomers nearby the three-fold axis arising in the NEP, respectively

According to our maps, NEP is slightly expanded compared with mature virion (157 vs. 152 Å in radius, ~3% expansion) (Fig. 2a). The capsid of NEP is thinner than that of mature virion (Fig. 2a), likely due to the expansion of NEP. Compared with the mature virus, NEP exhibits a 4.8° counter-clockwise rotation of the protomeric building block (VP1, VP0, and VP3) pivoting about the corner of VP3 at the icosahedral three-fold axis (Fig. 2b), and the VP1 was pushed away from quasi-three-fold axis region exhibiting a 6 Å shift towards the five-fold axis (Fig. 2b), leading to enlarged spacing between protomers (Fig. 2c, d). Specifically, the two-fold axis channel is markedly opened in the NEP than in mature virion (Fig. 2c, d, indicated by dashed rectangles in Fig. 2e, f). A second channel arises nearby the quasi-three-fold axis at the base of the canyon of NEP (indicated by dashed circles in Fig. 2e, f). In mature virion, this channel is blocked by the VP1 GH-loop, a VP1 fragment comprising residues 60–70, and the VP3 GH-loop (Fig. 2e, f). In addition, a slightly wider ditch is formed at the VP2/VP3 interface between adjacent protomers nearby the three-fold axis in the NEP (indicated by dashed ovals in Fig. 2e, f). Apart from these considerable altered overall surface properties, the interface areas between capsid proteins within pentamers or between pentamers are significantly reduced in CV-A10 NEP (Supplementary Table S2), suggesting a more dynamic nature of NEP.

A condensed RNA core surrounded by a loose RNA shell can be observed within the capsid of CV-A10 mature virion by low-pass filtering this map to 5 Å resolution (Supplementary Fig. S2a). The major densities bridging the capsid shell and the RNA shell underneath the three-fold symmetrical axis region and pentamer were identified (Supplementary Fig. S2a, b), and the possible residues involved in capsid interaction with RNA could be Arg43 and Arg62 of VP4, Arg18 of VP1, and to a less extent, Ser10 of VP2 (Supplementary Fig. S2b). Electrostatic potential analysis of the inner surface of pentamers revealed dominant clustering of positively charged residues in CV-A10 mature virion, including most of the residues involved in the bridge formation with the negatively charged RNA (Supplementary Fig. S2c), whereas these clustered positively charged patches were attenuated in NEP (Supplementary Fig. S2c), suggesting that the electrostatic potential might be one of the driving forces for RNA genome encapsulation/packaging.

### The “canyon” region of the CV-A10 mature virion

**Fig. 3:**
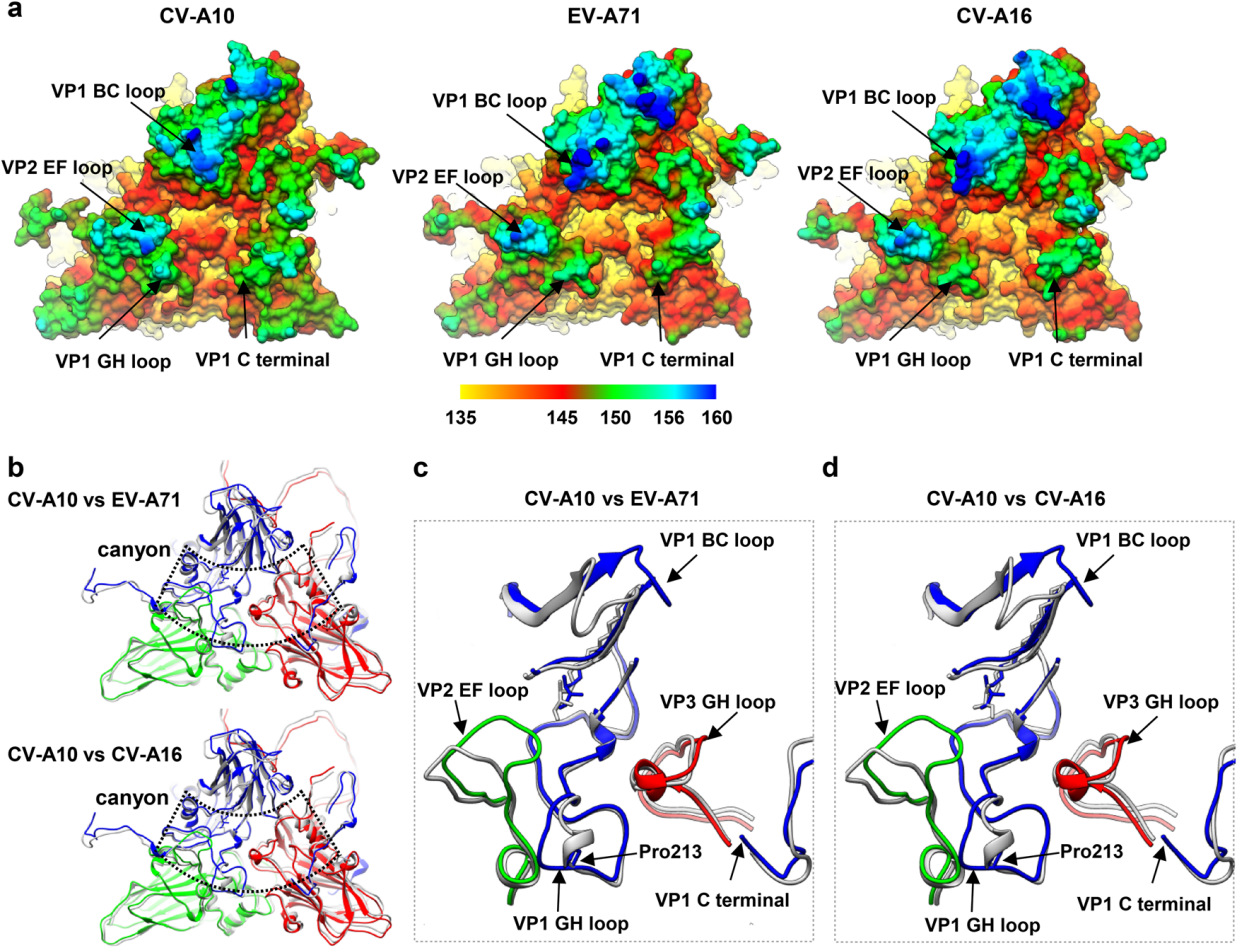
Comparison of canyon region among CV-A10, CV-A16, and EV-A71. **a** Molecular surfaces of one asymmetric unit plus an adjacent VP1 within a pentamer of CV-A10 mature virion, EV-A71 (3VBF), and CV-A16 (5C4W) visualized from outside of the capsid. The surface is radially colored as indicated by the color bar. **b** Model alignment of one asymmetric unit and the C-terminus of an adjacent VP1 within a pentamer of CV-A10 mature virion with that of EV-A71(3VBF) (upper panel), or CV-A16 (5C4W) (lower panel). The canyon location is indicated by dashed line. **c**, **d** Zoomed-in view of the major differences in the exposed loop regions surrounding the canyon between CV-A10 and EV-A71 (**c**) or between CV-A10 and CV-A16 (**d**). The models of EV-A71 and CV-A16 are in gray, and that of CV-A10 mature virion in color. For CV-A10 mature virion, the BC loop, GH loop, and C-terminus of VP1, EF loop of VP2, and GH loop of VP3 are indicated by black arrows. Pro 213 of VP1 in CV-A10 is also indicated by a black arrow

For some picornaviruses, the “canyon” circulating the five-fold axes is the location where receptors with an immunoglobulin-like fold insert into^19^. It has also been shown that the “canyon” region is involved in binding of non-immunoglobulin-like receptors for some enteroviruses^19,27^. Similar to other enteroviruses, CV-A10 virion also displays the canyon feature surrounding the five-fold axis (Fig. 1a, b), which appears to be shallower and narrower than that in EV-A71, CV-A16, and poliovirus type 1 (PV-1) (Fig. 3a and Supplementary Fig. S3). This is probably because the CV-A10 VP1 BC-loop at the northern rim of the canyon moves inwards whereas the VP2 EF-loop and the VP1 C-terminus at the south rim extends towards the canyon (Fig. 3a).

The surface-exposed loops that form the rims of the canyon are the most important neutralizing immunogenic sites of enteroviruses^28–32^. Several surface loop regions around the canyon of CV-A10 are significantly different from those of EV-A71 and CV-A16, not only in amino acid sequence (Supplementary Fig. S4) but also in conformations (Fig. 3b–d). Specifically, the VP1 BC loop shows obviously distinct conformations in CV-A10 compared with those in EV-A71 and CV-A16, and the VP1 GH loop of CV-A10 does not have the typical small α-helix as in EV-A71 and CV-A16, but forms a loop (Fig. 3c, d), probably due to the presence of Pro213 in the VP1 GH loop of CV-A10, which may have disrupted the α-helix formation. To a lesser degree, the VP3 GH-loop adopts a slightly more expanded conformation in CV-A10 than that in EV-A71 or CV-A16 (Fig. 3c, d). In addition, the VP2 EF loop is bended and adopts a very distinct conformation in CV-A10 (Fig. 3b–d). Taken together, these loops shape a different canyon environment in CV-A10 compared with that of EV-A71 and CV-A10.

### Pocket factor within the CV-A10 virion

**Fig. 4:**
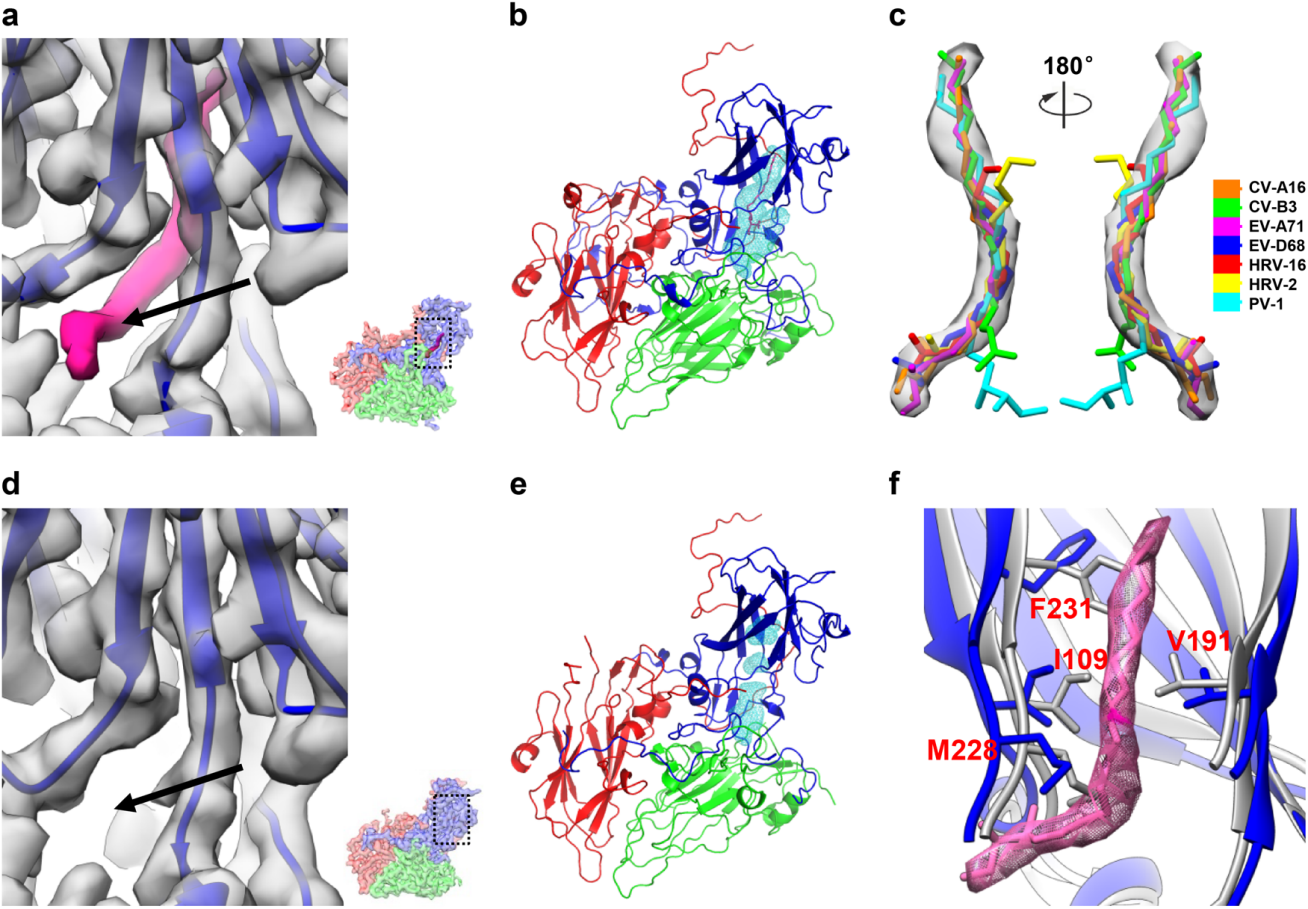
VP1 pocket region and identification of pocket factor in the CV-A10 mature virion. **a** Zoomed view of the VP1 pocket region in CV-A10 mature virion. The pocket location with respect to the complete CV-A10 protomer is illustrated in a small panel in the lower-right corner. The density corresponding to the pocket factor is highlighted in hot-pink. The entrance of the pocket is indicated by a black arrow. **b** The hydrophobic pocket (cyan mesh) in VP1 of CV-A10 mature virion is occupied by a natural lipid (hot-pink). **c** Fitting of putative pocket factor structures from seven known enterovirus structures (CV-A16: 5C4W, CV-B3: 1COV, EV-A71: 3VBF, EV-D68: 4WM8, HRV-16: 1AYN, HRV-2: 1FPN, and PV-1: 1VBD) into the corresponding density in the cryo-EM map of CV-A10 mature virion. **d** Zoom-in view of the VP1 pocket region in CV-A10 NEP. The same visualization style as in (**a**) was adapted. No density corresponding to the pocket factor can be observed. **e** The empty, collapsed pocket (cyan mesh) of CV-A10 NEP. **f** Comparison of the pocket region between CV-A10 mature virion (blue) and NEP (gray). Here the density and atomic model corresponding to the pocket factor are shown in hot pink

Enteroviruses possess a hydrophobic “pocket” lying beneath the “canyon”^9,10,19^. It is often occupied by a host cell-derived lipidic “pocket factor” that stabilizes the virion^9,10,19^. In our CV-A10 mature virion map, density corresponding to a pocket factor can be clearly observed inside the “pocket” (Fig. 4a). Fitting the available pocket factor structures of picornaviruses into the corresponding density of our 2.84 Å resolution mature virion cryo-EM map revealed that the pocket factor density of CV-A10 has the highest resemblance with that of EV-A71 (PDB 3VBF, Fig. 4b, c), corresponding to a fatty acid with an aliphatic chain of about 18 carbon atoms^11,33^. The pocket factor of CV-A10 has about 40 Å^2^ of surface accessible to solvent (Fig. 4a), which is similar to that of EV-A71^11,12^ and CV-A16^13^. These observations are in line with the highly conserved primary sequence lying in the VP1 pocket and similar VP1 pocket environment among CV-A10, EV-A71, and CV-A16 (Supplementary Table S3 and Supplementary Fig. S5a–c).

No density corresponding to the pocket factor was observed in the CV-A10 NEP map (Fig. 4d). In fact, the hydrophobic pocket is collapsed in the NEP compared with that in the mature virion (Fig. 4b, e). The volume of the pocket is significantly reduced because four residues (Ile109, Val191, Met228, and Phe231 of VP1) move inward (Fig. 4f), making it impossible to accommodate the pocket factor.

### Structure-guided high-throughput screening of pocket-binding compounds

Some small hydrophobic molecules can bind into the hydrophobic pocket of a variety of enteroviruses, leading to inhibition of corresponding enterovirus infections^20,21^. One of such pocket-binding inhibitors, namely pleconaril, showed good efficacy in treating picornavirus-caused respiratory illness in clinical trials^22^. Thus, we investigated whether pleconaril could also inhibit CV-A10 infection. Two other well-characterized pocket-binding inhibitors, WIN51711^34^ and Pirodavir^35,36^, were also evaluated in parallel with pleconaril. Surprisingly, based on cytopathic effect (CPE) observation, none of the three compounds (pleconaril, pirodavir, and WIN51711) exhibited inhibitory effect against the three CV-A10 strains (Kowalik, S0148b, and S0273b) even at the highest concentration tested (100 μg/ml) (Supplementary Table S4), despite they showed inhibitory effect against other enteroviruses, including EV-A71, CV-A16, and EV-D68 (Supplementary Table S4), at levels comparable with those reported previously^33,35,37–39^. Structural modeling showed that there are potential clashes between the compounds and key residues lining the VP1 pocket of CV-A10 (Supplementary Fig. S6), which may prohibit binding of these compounds into the VP1 pocket of CV-A10, resulting in the lack of inhibitory effect on CV-A10. This clash may be related to several variations of amino acids and conformations in the highly conserved region of VP1 pocket among CV-A10, EV-A71, CV-A16, and EV-D68 (Supplementary Fig. S6 and Supplementary Table S3).

**Fig. 5:**
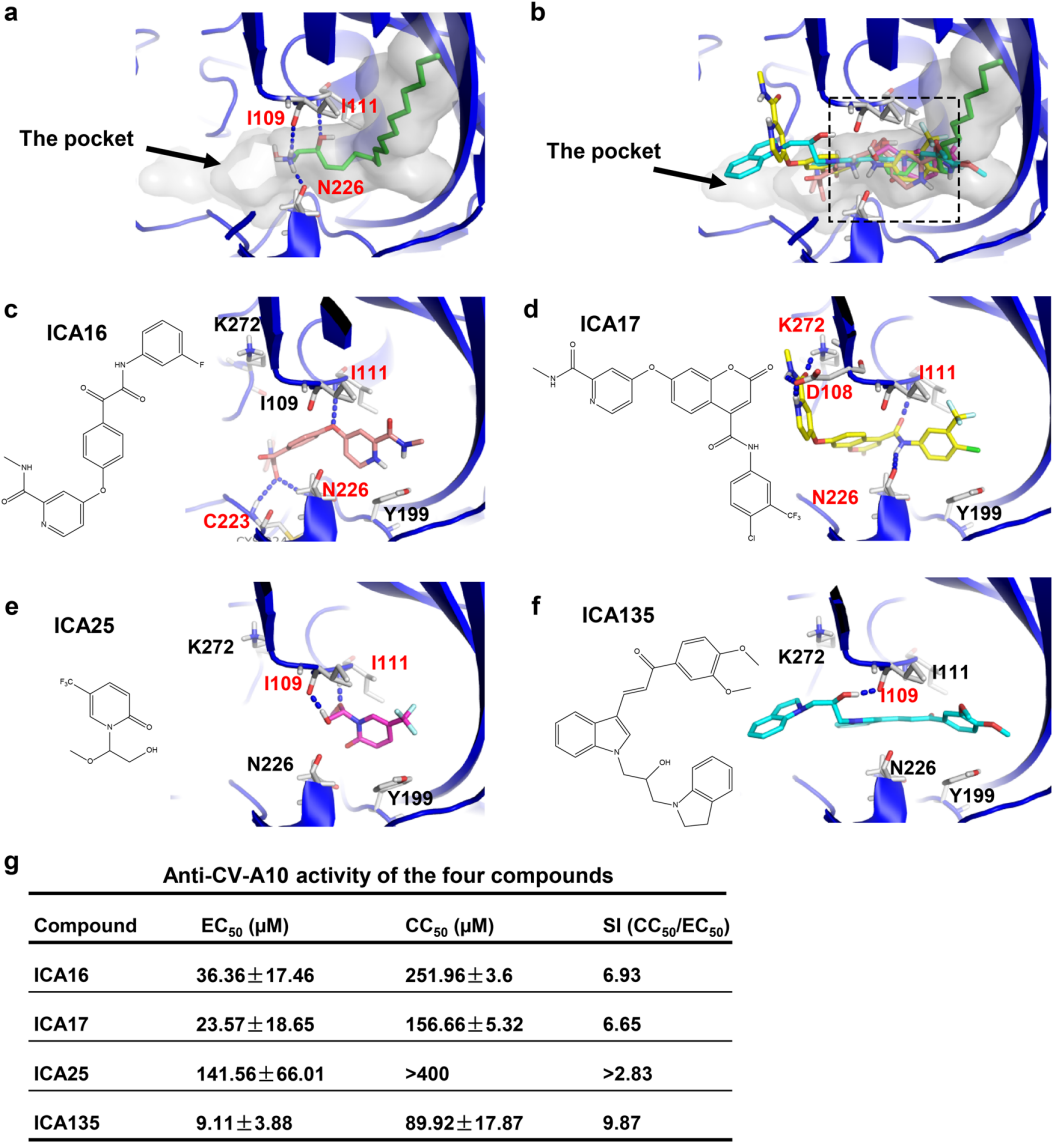
Binding modes of SPH and the four pocket-binding compounds and the anti-CV-A10 activity measurement. **a** The pocket factor of CV-A10, SPH, occupies part of the pocket (shown as transparent surface) and forms hydrogen bonds with I109, I111, and N226 of VP1 in CV-A10. Hydrogen bonds are shown as blue dashed lines. **b** Predicted binding positions of the four compounds in VP1 pocket of CV-A10 mature virion. The pocket (transparent surface) and the pocket factor are also shown to illustrate the relative locations of the four compounds. The four compounds are shown as sticks and colored by elements, with the carbon atoms of ICA16, ICA17, ICA25, and ICA135 colored in salmon, yellow, magenta, and cyan, respectively. The overlapping region inside the pocket among the CV-A10 pocket factor and the four compounds is indicated by the dashed rectangle. **c**–**f** Binding modes of ICA16 (**c**), ICA17 (**d**), ICA25 (**e**), and ICA135 (**f**) to the VP1 pocket of CV-A10 mature virion predicted by docking analysis (using Glide 6.9 in its SP mode). CV-A10 is shown in blue cartoon, and the compounds in stick. Potential hydrogen bonds formed between the pocket and the compounds are shown as blue dashed lines, and the residues involved in the hydrogen bond formation are labeled in red. **g** List of EC50, CC50, and SI (CC50/EC50) values of the four compounds against CV-A10 (S0148b). 50 µl of serially diluted compounds was mixed with 50 µl of CV-A10 virus containing 100TCID50. The mixtures were incubated for 1 h and then added to 2 × 10^4^ RD cells, followed by incubation at 37 °C for 48 h. Then, culture supernatants were collected and analyzed for virus titers by plaque assays. Each experiment was performed at least three times

To search for a potent anti-CV-A10 pocket-binding inhibitor, we firstly performed structure-based high-throughput virtual screening. According to our mature virion cryo-EM structure, the pocket factor of CV-A10 is mostly buried in the hydrophobic pocket of VP1, and its main body forms abundant hydrophobic interactions with surrounding residues, whereas its head forms hydrogen bonds with residues near the entrance and middle position of the pocket, including Ile109, Ile111, and Asn226 (Fig. 5a). We virtually screened about 4 million small compounds by calculating whether they can bind into the VP1 pocket region of this CV-A10 mature virion structure. After the virtual screening, 258 compounds with high docking scores were selected for further evaluation by cell-based in vitro inhibition assays. Four out of these compounds, designated ICA16, ICA17, ICA25, and ICA135 (Fig. 5b–f and Supplementary Table S5), were found to be able to inhibit CV-A10 infection. The half-maximal effective concentration (EC50) and cytotoxic concentration (CC50) values of these four compounds were shown in Fig. 5g. The in silico docking models showed that the binding sites of the four compounds are all located near the entrance and middle part of the VP1 pocket, partially overlapping with that of the pocket factor of CV-A10 (Fig. 5b–f, indicated by dashed rectangle in Fig. 5b). Several VP1 residues, such as Ile109, Ile111, Asn226, and to a less extent, Asp108, Lys272, and Cys224, likely play an important role in the interactions between the compounds and the pocket (Fig. 5c–f). In particular, ICA135 could potentially form hydrogen bonds with residue Ile109 and all the other three compounds could potentially form hydrogen bonds with residue Ile111 (Fig. 5c–f). It is worth noting that Ile109 and Ile111, which are located near the entrance and middle position of the VP1 pocket, are also involved in the formation of hydrogen bonds with the pocket factor of CV-A10 (Fig. 5a).

### In vitro and in vivo inhibitory effect of ICA135 against CV-A10 infection

**Fig. 6:**
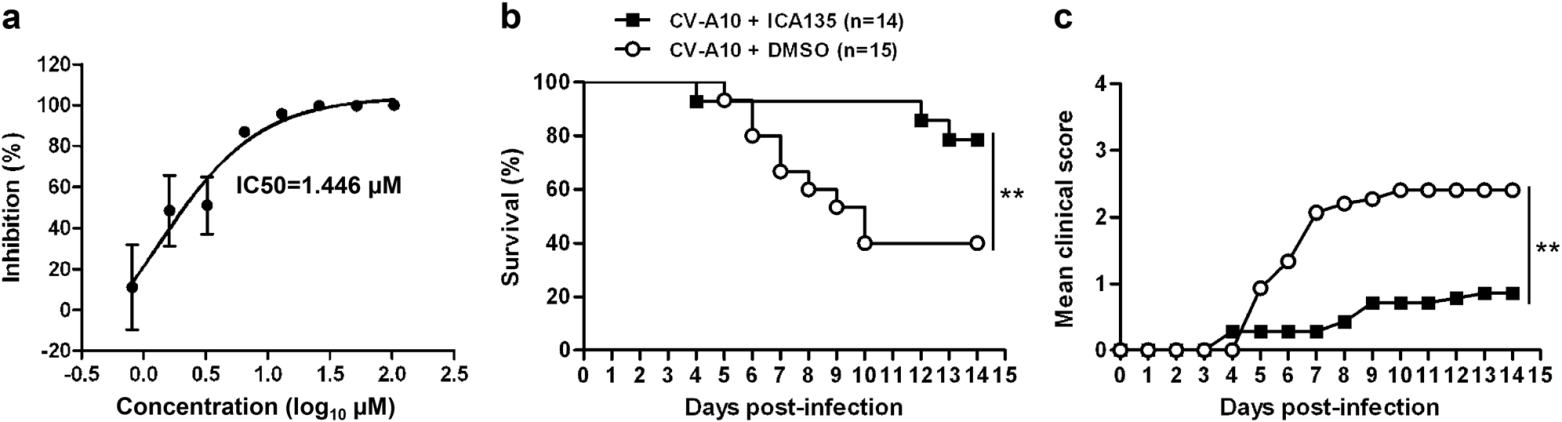
In vitro and in vivo inhibitory activities of ICA135 against CV-A10 infection. **a** Inhibitory effect of ICA135 on CV-A10 infection of RD cells. 50 µl of CV-A10/S0148b virus containing 100 TCID50 was mixed with 50 µl of compound dilutions, and incubated at 37 °C for 1 h. The mixtures were added to 2 × 10^4^ RD cells to allow infection at 37 °C for 6 h. Then, the mixtures were replaced with fresh medium and the plates were incubated at 37 °C for 24 h. The cells and medium were harvested and subjected to realtime PCR assay. Each experiment was performed at least three times. **b**, **c** In vivo inhibitory effect of ICA135. Groups of 7-day-old ICR mice (*n* = 14 or 15) were inoculated intraperitoneally (i.p.) with 8 × 10^4^ TCID50 of CV-A10/S0148b in the absence or presence of 50 mg/kg body weight of ICA135. Then the mice were monitored daily for survival (**b**) and clinical signs (**c**) for a period of 14 days. Clinical scores were graded as follows: 0, healthy; 1, reduced mobility; 2, limb weakness; 3, paralysis; 4, death. Survival curves for the control and treatment groups were compared by Log-rank test using GraphPad Prism software. Mean clinical scores for the two groups were compared by two-way ANOVA test. Statistical significance was indicated as **P ≤ 0.01

Based on the results from the virus yield reduction assays (Fig. 5g), ICA135 appeared to be the most potent among the four identified inhibitors. To measure its inhibitory activity, we further analyzed CV-A10 replication in the absence or presence of different concentrations of ICA135 by qPCR assay. As shown in Fig. 6a, ICA135 inhibited CV-A10 replication in a dose-dependent manner with the half-maximal inhibitory concentration (IC50) being 1.446 µM.

The in vivo inhibitory effect of ICA135 was assessed in an established mouse model of CV-A10^24,40^. The control mice (*n* = 15) that were infected with mock (DMSO)-treated CV-A10 gradually developed clinical signs, such as limb weakness and paralysis, and 60% of them died by the end of the 14-day observation period (Fig. 6b, c). On the contrary, the majority (78.6%) of the mice inoculated with ICA135-treated CV-A10 (*n* = 14) was free of disease throughout the 14-day period, and in two of the three fatal cases, the occurrence of death was much delayed (12 and 13 dpi, respectively) as compared with that in the control group (5–10 dpi) (Fig. 6b, c). Clearly, the survival rate of the ICA135 treatment group was significantly (*P* ≤ 0.01) higher than that of the mock control group (Fig. 6b). These results show that ICA135 exerts protective function in vivo.

### ICA135 is a broad-spectrum inhibitor for human enteroviruses

**Fig. 7:**
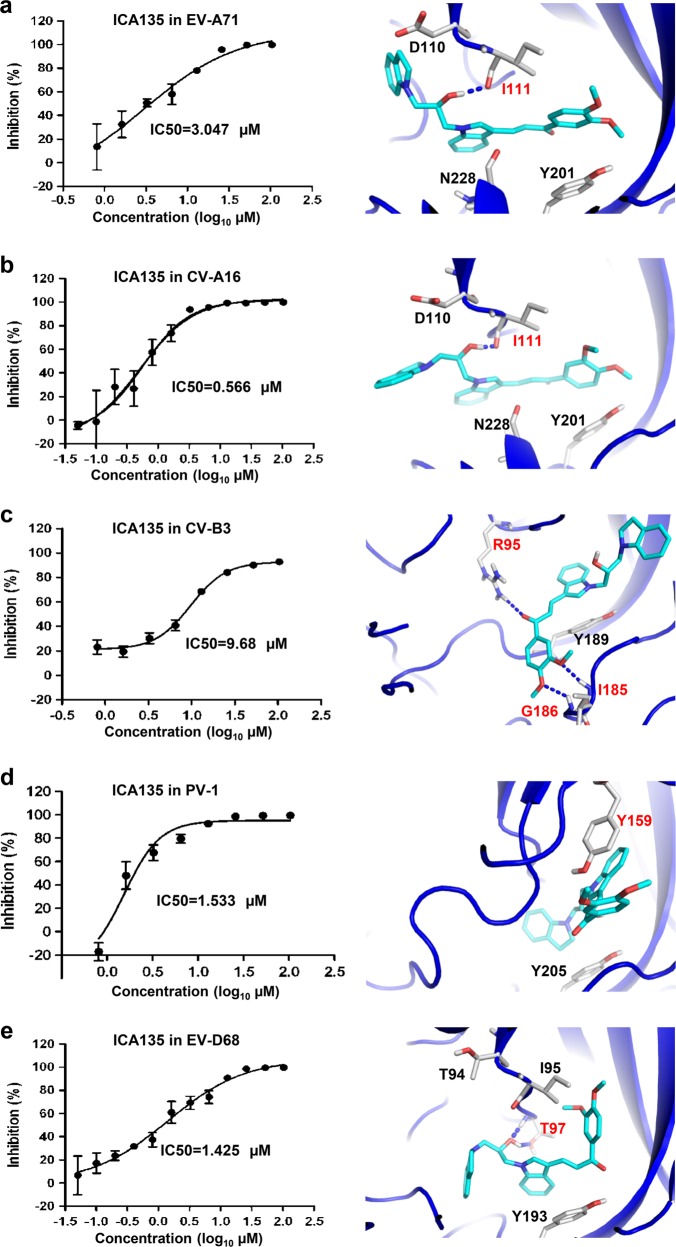
Broad-spectrum anti-enterovirus activity of ICA135. **a**–**e** ICA135 was tested for its in vitro inhibitory activity against a panel of enteroviruses, including EV-A71 strain G082 (**a**), CV-A16 strain SZ05 (**b**), CV-B3 strain Nancy (**c**), PV1 strain Sabin (**d**), and EV-D68 strain US/MO/14-18947 (**e**). Data shown in the left panels are representative results from at least two independent experiments for each virus. Binding modes of ICA135 into the corresponding viruses (right panels) were predicted by docking analysis. EV-A71 (PDB 3VBF), CV-A16 (PDB 5C4W), EV-D68 (PDB 4WM8), CV-B3 (PDB 1COV), and PV-1 (PDB 1VBD) are shown in blue cartoon with ICA135 in stick. Potential hydrogen bonds are shown as blue dashed lines. The residues involved in the hydrogen bond formation or π–π stacking are labeled in red

Enteroviruses infecting humans are categorized into four species, A, B, C, and D. Knowing that ICA135 is an inhibitor for CV-A10, we asked whether it also possesses inhibitory ability against other human enteroviruses. Representative viruses from the four species were tested, including EV-A71 and CV-A16 from specie A, CV-B3 from specie B, PV-1 from specie C, and EV-D68 from specie D. Results from the in vitro infection-inhibition assays showed that ICA135 exhibited inhibitory effect on all tested viruses with IC50s ranging between 0.566 and 9.68 µM (Fig. 7a–e, left panels). These data are in very well agreement with the results from in silico docking analysis, which showed that ICA135 could potentially bind to the VP1 hydrophobic pockets of EV-A71, CV-A16, CV-B3, PV-1, and EV-D68 with high-binding affinity (Fig. 7a–e, right panels and Supplementary Table S5). Although these tested viruses belong to different human enterovirus species, they still share broadly similar primary sequence lying the VP1 pocket and overall shape of VP1 hydrophobic pocket with CV-A10 (Supplementary Fig. S5d–f and Supplementary Table S3) and thus all of them could provide a suitable environment for the binding of ICA135. Putting together, the above results demonstrate that ICA135 is a broad-spectrum inhibitor for human enteroviruses.

## Discussion

In the present study, we determined the cryo-EM structures of CV-A10 mature virion and NEP at 2.84 and 3.12 Å, respectively, providing atomic resolution structural information for CV-A10. Our structures reveal that CV-A10 NEP is slightly expanded compared with the mature virion and displays opened two-fold axis channel and quasi-three-fold axis channel, comparable to those of EV-A71^11,12^. It is noteworthy that the NEPs of both CV-A10 and EV-A71 do not possess a pocket factor that has been shown to stabilize virions for a number of enteroviruses^9,10,19^. In contrast, the NEP of closely related CV-A16 contains a sphingosine-like pocket factor and related to this, no expansion was observed^13^. Therefore, lack of a pocket factor may account for the NEP expansion in CV-A10 and EV-A71. Besides, the rotation and movement of the protomeric building block of NEP in comparison with mature virion observed in CV-A10 may also contribute to the NEP expansion and thinner capsid of NEP.

The overall structures of CV-A10 are similar to those of EV-A71, CV-A16, and poliovirus. Like in other enteroviruses, a canyon, which is often the site that receptors bind to initiate the subsequent uncoating process^9,10,19^, also exists in CV-A10. Structure comparison shows that the canyon of CV-A10 is broadly similar to that of EV7-A1 and CV-A16, but is obviously shallower and narrower than that of poliovirus (Fig. 3a and Supplementary Fig. S3). The deep canyon on poliovirus surface allows penetration by the poliovirus receptor CD155, a molecule with immunoglobulin folds^41,42^. In contrast, the much reduced canyon space in EV-A71 and CV-A16 may not be able to accommodate the insertion of an immunoglobulin-like receptor. Indeed, human scavenger receptor class B member 2 (SCARB2), identified as the uncoating receptor for EV-A71 and CV-A16^43,44^, does not contain an immunoglobulin fold. The similarity in canyon structure between CV-A10 and EV-A71/CV-A16 suggests that CV-A10 may also use a nonimmunoglobulin-like molecule as its uncoating receptor. In agreement with this hypothesis, KREMEN1, which was recently identified as a receptor for CV-A10^45^, does not contain immunoglobulin-like domains^46^. Surface loops that shape the walls of the canyon are important for the interactions between enteroviruses and their receptors^19^. For example, the VP1 GH-loop of EV-A71 has been implicated to act as an adaptor-sensor for receptor binding^12^, which is supported by the findings that a peptide comprising the residues of this loop was capable of binding soluble SCARB2^47^ and monoclonal antibodies targeting this loop could block the interaction between EV-A71 and soluble SCARB2^30^. In this study, we showed that for CV-A10 some major surface loops around the canyon, in particular the VP1 GH loop, adopt conformations distinct from their counterparts in EV-A71 and CV-A16 (Fig. 3b–d). This may to some extent explain why CV-A10, unlike EV-A71 and CV-A16, could not utilize SCARB2 as its receptor^44,45^.

Surface loops on enteroviral capsids are often antigenic sites and contribute greatly to induction of neutralizing antibodies. Previous studies showed that the EF-loops and GH-loops of VP1, the EF-loop of VP2, and the knob and GH-loop of VP3 are neutralizing epitopes on EV-A71^30,48–51^, whereas CV-A16 contains neutralizing epitopes in the VP1 BC-loop, EF-loop, and GH-loop regions^32^. Neutralizing epitopes on CV-A10 have not yet been defined. Although the overall mature virion structures are similar among EV-A71, CV-A16, and CV-A10, the primary sequence and the structure of the aforementioned surface loops in CV-A10 are apparently different from those in EV-A71 or CV-A16 (Supplementary Fig. S4 and Fig. 3b–d). This suggests that antibodies targeting the surface loops of CV-A10 are unlikely able to cross-neutralize EV-A71/CV-A16, or vice versa. Indeed, our recent work showed that the antisera from mice immunized with an inactivated whole-virus CV-A10 experimental vaccine could not neutralize EV-A71 or CV-A16^24^. Additionally, the antisera induced by EV-A71 or CV-A16 experimental vaccines did not exhibit neutralization effect on CV-A10 ^52-54^. Hence, a vaccine targeting CV-A10 should be developed, which may ultimately be combined with immunogens derived from other HFMD-causing enteroviruses, such as EV-A71 and CV-A16, to formulate a multivalent vaccine with broad protective spectrum against HFMD.

In most enteroviruses, there is a natural lipidic pocket factor in the hydrophobic VP1 pocket, which functions to stabilize virions. Expulsion of the pocket factor after virus binding to its receptor is a prelude to the uncoating process required for infection^9,10,19^. Replacing the pocket factor with a high-affinity pocket-binding molecule may prevent viral uncoating and subsequent infection, and is therefore one of the strategies for anti-enteroviral drug development^20,21^. A number of such capsid-binding molecules, including WIN51711^33^, pleconaril^39^, and pirodavir^38^, have been shown to exhibit inhibitory effects on EV-A71. Our CV-A10 mature virion structure revealed the presence of a pocket factor corresponding to a fatty acid with 18 carbon atoms within the VP1 hydrophobic pocket (Fig. 4a–c). The CV-A10 pocket factor is highly similar to that in EV-A71 (Fig. 4c). However, cell-based inhibition assays showed that pleconaril, pirodavir, and WIN51711 are not effective for CV-A10 (Supplementary Table S4), suggesting that they may not be able to bind into the VP1 pocket of CV-A10. In consistence with this observation, in silico docking showed that the VP1 pocket in CV-A10 could not accommodate WIN51711, pleconaril, or pirodavir due to steric hindrance imposed by some VP1 residues including Phe133, Met193, and Asn226 (Supplementary Fig. S6).

Our atomic resolution CV-A10 mature virion structure allowed us to perform virtual screens for anti-CV-A10 capsid binders from about 4 million compounds, leading to a rapid identification of 258 candidate inhibitors. Eventually, four of them (ICA16, ICA17, ICA25, and ICA135) were verified to be able to inhibit CV-A10 infection in vitro. Examination of the predicted docking models (Fig. 5b–f) revealed that each of the four compounds binds well to the VP1 pocket of CV-A10. Apart from numerous surrounding hydrophobic residues which could interact with and stabilize these compounds, several VP1 residues, such as Ile109, Ile111, Asn226, and to a less extent, Asp108, Lys272, and Cys224, appeared to be critical for the interactions between inhibitors and the pocket (Fig. 5c–f). Nonetheless, the exact binding modes for these new inhibitors remain to be verified probably by determining structures of CV-A10 in complex with each of the inhibitors. It is noteworthy that, according to the docking prediction, the four ICA compounds do not fully occupy the space of the pocket factor, rather, they bind into the entrance and middle region of VP1 pocket and take up the space owned by the head and middle part of the pocket factor (Fig. 5b–f). This potential binding pattern of the four anti-CV-A10 inhibitors is distinct from those of WIN51711, pleconaril, and pirodavir determined in previous structural studies^33,37,55^, suggesting that the four anti-CV-A10 compounds may represent a new class of pocket-binding inhibitors. Significantly, ICA135, the most potent one among the four compounds, also exhibits comparable inhibitory effect against representative viruses from human enterovirus species A, B, C, and D (Fig. 7). It is noteworthy that the pocket factors of these viruses are of different length (18-carbon atom in CV-A10, EV-A71, and CV-A16, 14-carbon atom in CV-B3 and PV-1, and 10-carbon atom in EV-D68), suggesting that the effectiveness of ICA135 is independent of the length of the pocket factor or the species of enteroviruses. Rather, the unique ICA135-binding site (nearby the entrance and middle region of VP1 pocket), which is highly conserved among the enteroviruses tested (Supplementary Fig. S5 and Supplementary Table S3) and less affected by the collapse of VP1 pocket during viral entry, may contribute predominantly to the observed broad-spectrum anti-enterovirus inhibition by ICA135. Clearly, ICA135 is a lead compound exhibiting broad-spectrum inhibitory effects on the major HFMD-causing type A enteroviruses and other representative viruses from the other three species of human enteroviruses, and can be used as a scaffold for further development of more potent inhibitors.

In summary, our study elucidates atomic structures of CV-A10, thereby providing structural basis for design and development of anti-CV-A10 vaccines and therapeutics. Our structure-guided high-throughput drug screening identifies a lead compound, which potentially binds to the highly conserved entrance and middle region of VP1 pocket and shows inhibitory effects on a number of viruses covering all four species (A–D) of human enteroviruses. Our study should facilitate the development of broad-spectrum-inhibiting drugs for human enteroviruses, especially those causing HFMD.

## Materials and methods

### Cells and viruses

RD and Vero Cells were maintained in Dulbecco’s modified Eagle’s medium (DMEM) containing 10% fetal bovine serum (FBS) as described previously^56,57^. CV-A10 strains S0148b, S0273b, and Kowalik have been described in a previous study^24^. EV-A71/G082 and CV-A16/SZ05 have been described previously^54^. EV-D68 strains US/MO/14-18947 (ATCC Number: VR-1823), US/KY/14-18953 (VR-1825), and Fermon (VR-1826) were obtained from ATCC. CV-B3 strain Nancy and PV-1 strain Sabin have been described in a previous study^58^. All virus stocks were made in RD cells. Virus titers were determined according to the Reed–Muench method^59^ and expressed as 50% tissue culture infectious dose (TCID50).

### Preparation of CV-A10 particles

CV-A10 (S0148b strain) was propagated on 80% confluent Vero cells with an MOI of 0.01. 3 days later, when ~90% of cells showed CPE, the cultures were harvested and subjected to three freeze–thaw cycles. The virus was treated with β-propiolactone (1:100,000, V/V) at 4 °C for 12 h. The remaining β-propiolactone was hydrolyzed by incubation at 37 °C for 2 h. After cell debris was removed, the virus containing supernatant was subjected to ultracentrifugation on a 20% sucrose cushion (SW28 Ti rotor, 131,000*g*, 4 °C, 3 h) and then on a 10–50% sucrose gradient (SW60 Ti rotor, 205,000*g*, 4 °C, 2.5 h) in a Beckman centrifuge. The virus was further purified by size-exclusion chromatography using Sephacryl S-500 column (GE Healthcare, USA). Purified CV-A10 samples were quantified by Bradford assay and analyzed by SDS–PAGE and Western blot assays.

### Cryo-EM imaging

An aliquot of 2.6 µl purified CV-A10 sample was deposited onto a glow discharged holey carbon Quantifoil Cu grid (R1.2 × 1.3, 200 mesh, Quantifoil Micro Tools), which was covered by a thin layer of homemade continuous carbon film. After 4 s blotting to remove extra sample, the grid was plunge-frozen into liquid ethane using a FEI Mark IV Vitrobot. Specimen was subsequently imaged in an FEI Titan Krios transmission electron microscope operated at 300 kV and equipped with a Cs corrector. Images were recorded on a K2 direct electron detector in super-resolution mode with a pixel size of 0.67 Å. Each movie was dose-fractioned into 38 frames. The total electron dose was set to ~38 e^−^/Å^2^ and the exposure time was 7.6 s. Defocus values for this dataset varied from −0.6 to −2.0 µm.

### Cryo-EM single particle 3D reconstruction

To correct the drift and beam-induced movement, the 38 frames in each movie were aligned and averaged to a single micrograph using MotionCorr^60^. Empty and filled particles were semi-automated boxed out separately using *e2boxer.py* program from EMAN2.1^61^. These particles were extracted with a pixel size of 1.005 Å by Relion1.3^62^ and then imported into jspr^25^. CTF fitting was automatically performed using *fitctf2.py* program in jspr^25^, then visually adjusted using EMAN1.9 *ctfit* program. After CTF correction, the particles were separated into two halves. For each half of the data, we independently performed reference-free 2D analysis and initial model building in EMAN2.1^61^. All the 3D reconstruction was performed using jspr package^25^ following the gold standard 3D reconstruction procedure. Further refinement of euler/center, defocus, astigmatism, beamtilt, and scale/anisoscale^63^ were also carried out in jspr^25^. A 2.84 Å resolution map of CV-A10 mature virion was reconstructed using 13,273 particles, and a 3.12 Å resolution map of CV-A10 NEP was reconstructed using 23,312 particles. The map resolutions were assessed using the gold standard criteria of 0.143 FSC cutoff^64^. Finally, the cryo-EM maps of CV-A10 mature virion and NEP were sharpened using a *B* factor of −109.4 and −125.6 Å^2^, respectively, and then low-pass filtered to the determined resolutions using *e2proc3d.py* in EMAN2.1.To further validate our two maps, we performed local-resolution evaluation by using *ResMap*^26^.

### Model building

Homologous crystal structures of EV-A71 mature virion (PDB: 3VBF) and empty particle (PDB: 3VBO) were chosen as templates to build the initial homologous models for our CV-A10 mature virion and NEP, respectively, through SWISS-MODEL website. Side-chain densities were clearly visualized throughout our two cryo-EM maps, which allowed us to fit the models into the corresponding maps very well. We then performed additional local manual refinement of the model against the map in several less well-fitted regions using COOT^65^. Finally, the entire atomic model were further automatically refined against the map using Phenix real-space refinement procedure by program *phenix.real_space_refine*^66^. The final model was validated using *phenix.molprobity*^67^; The validation statistics are shown in Supplementary Table S1.

Figures were generated with either UCSF Chimera^68^, or PyMOL (http://www.pymol.org).

### Structure-based virtual drug screening

The virtual screening of CV-A10-specific antivirals was carried out on about 4 million drugs which contain both synthesized compounds and commercial compounds from Specs, Chemdiv, and Enamine. These compounds were preprocessed by the Ligprep 3.6 program (Schrödinger, LLC, New York, NY, USA) applying OPLS_2005 force field before molecular docking, with Epik 3.4 (Schrödinger, LLC, New York, NY, USA) to generate the proper protonation states at pH 7.0 ± 2.0. A restrained minimization of the crystal structure was performed to reorient side-chain hydroxyl groups before further processing. The VP1 pocket of CV-A10 mature virion was selected to define and generate the receptor grid. In silico docking was performed by Glide 6.9 (Schrödinger, LLC, New York, NY, USA) in standard precision (SP) with default values for other parameters. The threshold value of docking score was identified as −7.84 kcal/mol, which was obtained by re-docking the pocket factor into the CV-A10 structure model. After careful visual evaluation of the docked conformations, 258 compounds were selected for further in vitro analysis and assessment. The same in-silico docking procedure was performed when docking ICA135 into EV-A71 (3VBF), CV-A16 (5C4W), EV-D68 (4WM8), CV-B3 (1COV), and PV-1 (1VBD).

### Compounds

Pleconaril was purchased from Toronto Research Chemicals (Toronto, Canada). Pirodavir was purchased from ToYongBio (Shanghai, China). WIN51711 was kindly provided by Shanghai Institute of Organic Chemistry, Chinese Academy of Sciences (Shanghai, China). Other compounds used in this study were provided by Shanghai Institute of Materia Medica, Chinese Academy of Sciences (Shanghai, China). All compounds were dissolved in DMSO to make stocks (10 mg/ml).

### Primary in vitro screening assay

For screening, each compound was diluted with DMEM medium to 100 and 50 µg/ml. Then, 50 µl of each compound dilution was mixed with equal volume of the indicated virus containing 100 TCID50 in wells of 96-well plates. The plates were incubated at 37 °C for 1 h. Then, 100 μl of cell suspension (containing 1.5 × 10^4^ RD cells) was added to each well, followed by incubation at 37 °C. After 72 h, the cells were observed to evaluate the appearance of CPEs. Compounds that were able to fully inhibit CPE at the 50 µg/ml concentration were selected for further inhibition and cytotoxicity analyses.

### Plaque assay

Vero cells were seeded in 24-well plates at 1 × 10^5^ cells per well in 500 µl of DMEM supplemented with 10% FBS, 100 U/ml penicillin and 100 µg/ml streptomycin, and incubated at 37 °C for 24 h. Virus samples were serially diluted 10-fold with DMEM containing 2% FBS. Then, 200 μl of virus dilutions was added to wells of 24-well plates containing preseeded Vero cells. The plates were incubated at 37 °C with 5% CO_2_ for 1 h, and then the virus inocula were replaced with 700 µl of DMEM plus 0.8% carboxymethylcellulose (Sigma, USA) and 2% FBS. After 3 days of incubation at 37 °C with 5% CO_2_, the cells were fixed with 4% paraformaldehyde and stained with 1% crystal violet, and plaques were counted.

### Determination of drug EC50 and CC50

The EC50s of selected compounds were determined by performing virus yield reduction assays. Briefly, 50 μl of serially diluted compounds was mixed with 50 μl of CV-A10 virus containing 100TCID50. The mixtures were incubated for 1 h and then added to 2 × 10^4^ RD cells, followed by incubation at 37 °C for 48 h. Then, culture supernatants were collected and analyzed for virus titers by plaque assays as described above. EC50s were calculated by regression analysis of the mean virus titers expressed as percentages of untreated, virus-infected control values for each concentration.

For determination of drug CC50, RD cells (2 × 10^4^ cells in 100 μl of DMEM containing 2% FBS) were seeded into each well of a 96-well plate and incubated at 37 °C with 5% CO_2_ for 24 h. A serial dilution of the selected compounds was added to the wells containing preseeded cells. Plates were incubated at 37°C for 48 h and then allowed to equilibrate to room temperature for 30 min. Then, 50 μl of CellTiter-Glo (Promega, USA) reagent was added to each well, and the plates were incubated at room temperature for 10–30 min before being read with a Thermo Varioskan™ Flash (Thermo, USA). The CC50s were calculated by regression analysis of the means of the luminescences expressed as percentages of untreated, uninfected control values for each concentration.

### In vitro inhibition assay and qPCR analysis

The ability of the selected compounds to inhibit virus replication in vitro was determined by qPCR analysis. Briefly, 50 µl of the indicated virus containing 100 TCID50 was mixed with 50 µl of compound dilutions, and incubated at 37 °C for 1 h. The mixtures were added to pre-seeded RD cells to allow infection at 37°C for 6 h. Then, the mixtures were replaced with fresh medium and the plates were incubated at 37 °C for 24 h. The cells and medium were harvested and subjected to total RNA extraction and reverse transcription as described previously^69^. The resultant first strand cDNA were used as template for quantitative real-time PCR (qRT-PCR) using the SYBR Premix Ex Taq^TM^ kit (Takara, Dalian, China) and Applied Biosystem 7900HT Real-time PCR system. Supplementary Table S6 lists the virus-specific primers used in this study. β-actin mRNA was also measured, serving as an internal control. Data analysis was performed using the 2^−ΔΔCt^ method as described previously^30^.

### Mouse study

The in vivo efficacy of ICA135 was evaluated in an established mouse model of CV-A10 infection^24,40^. Briefly, groups of 7-day-old ICR mice (14 or 15 animals/group) were inoculated intraperitoneally (i.p.) with 8 × 10^4^ TCID_50_ of CV-A10/S0148b in the absence or presence of 50 mg/kg body weight of ICA135. Then the mice were monitored daily for survival and clinical signs for a period of 14 days. Clinical scores were graded as follows: 0, healthy; 1, reduced mobility; 2, limb weakness; 3, paralysis; and 4, death. At the end of the 14-day observation period, all remaining mice were terminated by euthanasia. The in vivo virus challenge experiments were carried out in the biosafety level 2 (BSL2) animal facility at Institut Pasteur of Shanghai. This mouse study (protocol number: A2018039) was approved by IACUC at Institut Pasteur of Shanghai. Animals were cared for according to the institutional guidelines.

### Accession numbers

The final cryo-EM density maps of CV-A10 mature virion and NEP have been deposited in the Electron Microscopy Data Bank (EMDB) under accession codes EMD-9674 and EMD-9675, respectively. The atomic models of CV-A10 mature virion and NEP have been deposited in the Protein Data Bank (PDB) under accession codes 6IIJ and 6II0, respectively.

## Electronic supplementary material

Supplementary Information

## Electronic supplementary material

**Supplementary Information** accompanies the paper at (10.1038/s41421-018-0073-7).
