# Supplementary material for: Coxsackievirus A10 atomic structure facilitating the discovery of a broad-spectrum inhibitor against human enteroviruses
Source: Cell Discov. 2019 Jan 15;5:4. doi: 10.1038/s41421-018-0073-7 (PMC6331555; doi:10.1038/s41421-018-0073-7)
Supplement: Supplementary file 1 — Supplementary Information [file 41421_2018_73_MOESM1_ESM.pdf]

Supplementary Information

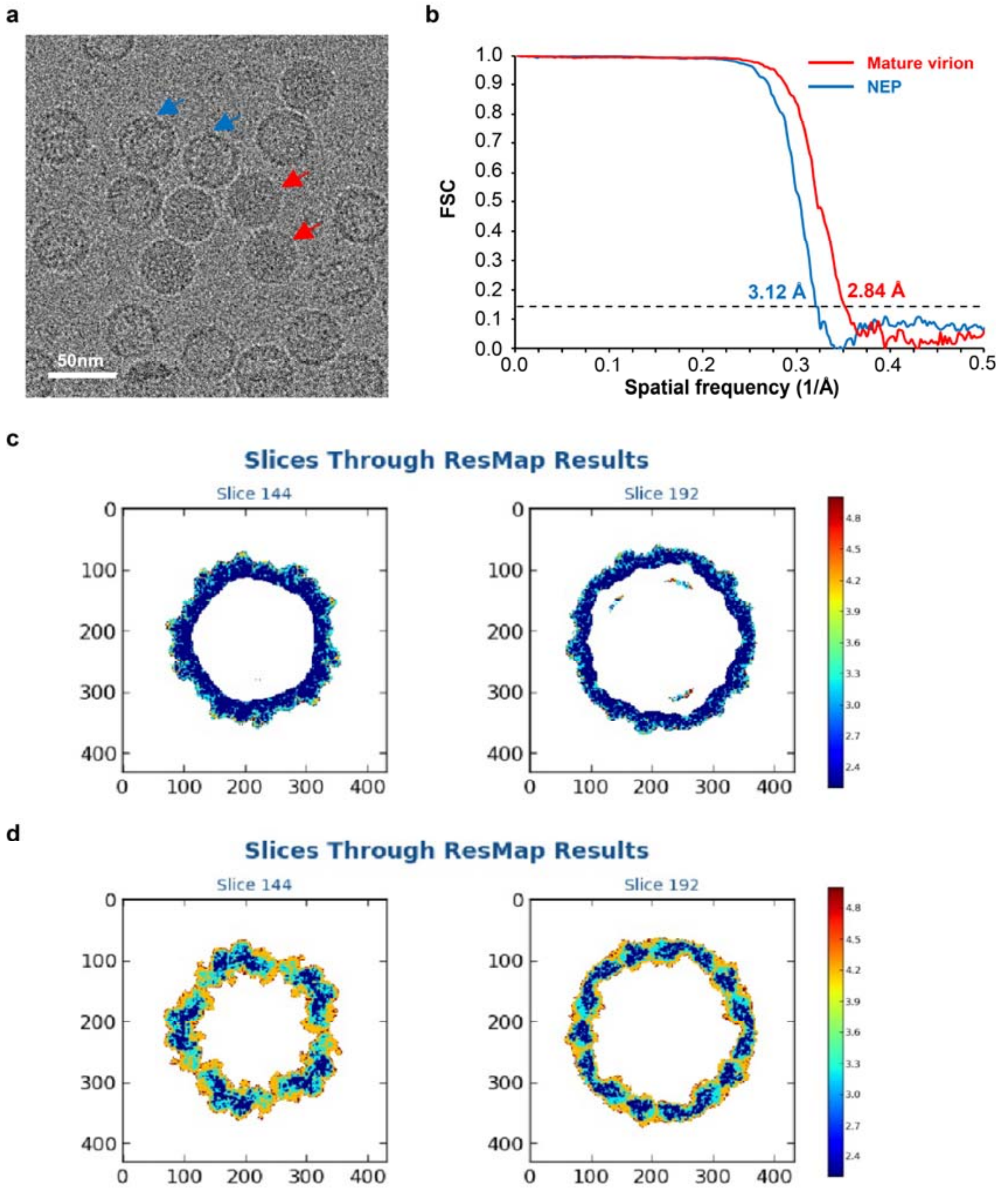

Supplementary Fig. S1. Cryo-EM image of CV-A10 and resolution evaluation of the cryo-EM map. **(a)** A representative cryo-EM image of CV-A10. The red and blue arrows indicate mature virion and NEP, respectively. Bar = 50 nm. **(b)** Resolution assessment of our two cryo-EM maps (CV-A10 mature virion and NEP) by Fourier shell correlation (FSC) at 0.143 criterion. **(c, d)** Local resolution evaluation of the CV-A10 mature virion **(c)** and NEP particle **(d)** maps estimated by Resmap. For each map, two representative discrete 2D slices were shown. The color bar on the right labels the corresponding resolution (unit is Å).

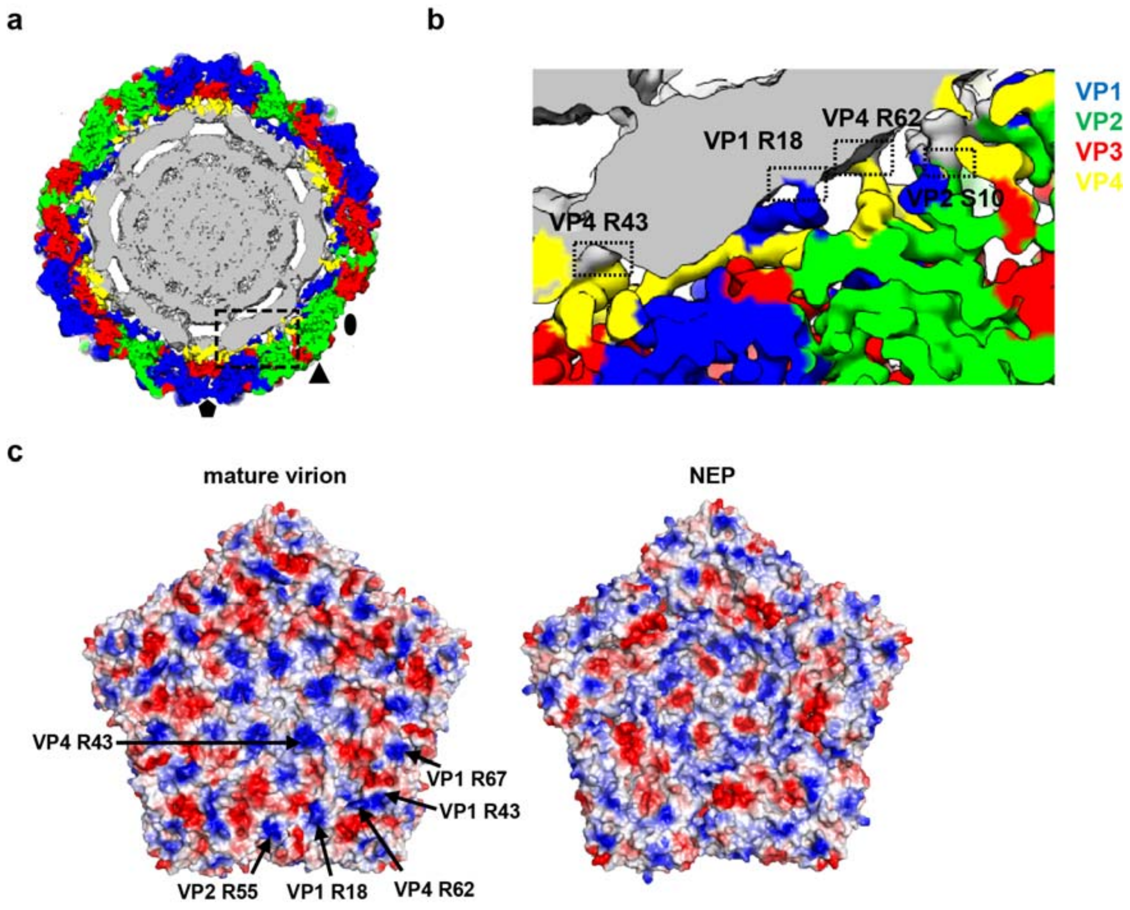

Supplementary Fig. S2. Genomic RNA interaction with CV-A10 capsid. **(a)** A central slice through the CV-A10 mature virion map, which was low-pass filtered to 5 Å resolution and viewed along the 2-fold axis. RNA is colored in gray. Black oval, triangle, and pentagon represent the 2-fold, 3-fold, and 5-fold axes, respectively, which style was followed throughout. **(b)** Close-up view of viral RNA–protein interaction interface, with the major bridges formed between viral RNA and protein capsid highlighted by dash line rectangles. **(c)** Electrostatic surface of a pentamer of CV-A10 mature virion and NEP, visualized from outside of the capsid. Positively charged amino acids were colored in blue, while negatively charged amino acids in red. The major amino acids contributing to the positive charges are indicated in CV-A10 mature virion.

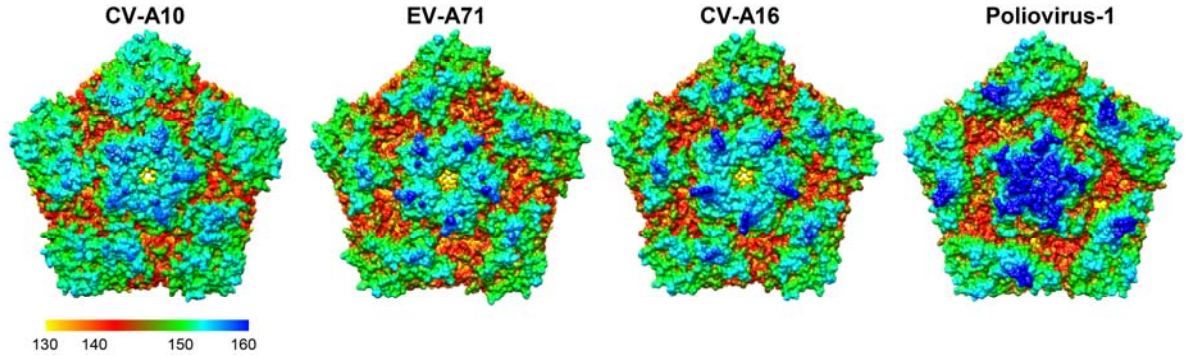

Supplementary Fig. S3. Molecular surfaces of pentamers from several enteroviruses. Radially colored molecular surfaces of one pentamer from CV-A10 mature virion, EV-A71(3VBF), CV-A16 (5C4W), and PV-1(1VBD), respectively.

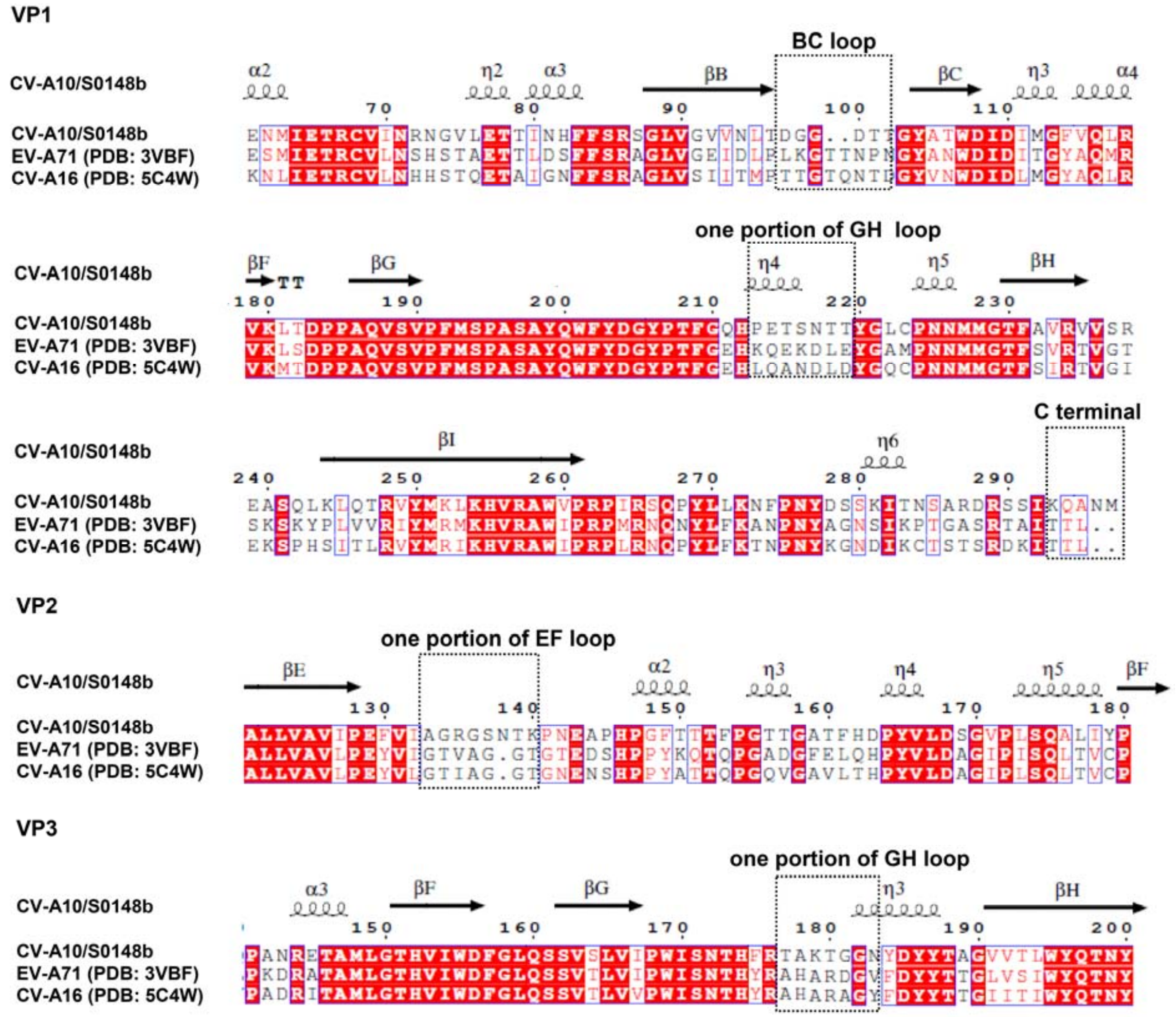

Supplementary Fig. S4. Sequence alignment of several loops around the canyon of CV-A10, EV-A71, and CV-A16. Espript representation of a structure-based sequence alignment of several fragments of CV-A10 (strain S0148b) VP1, VP2, and VP3 with that of EV-A71 (PDB 3VBF) and CV-A16 (PDB 5C4W), respectively. The sequence alignment indicates the sequence variations in the VP1 BC loop, VP1 GH loop, VP1 C terminal, VP2 EF loop, and VP3 GH loop among these three strains. The secondary structural elements for CV-A10 are also shown at top.

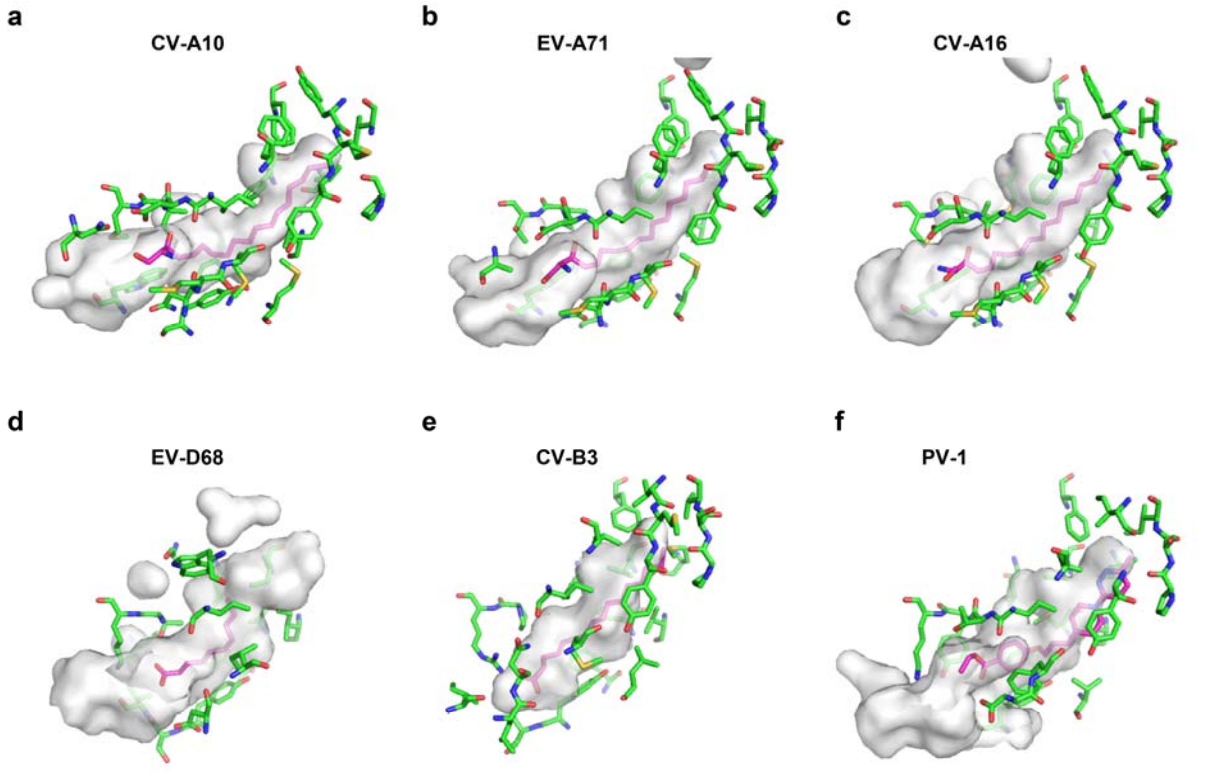

Supplementary Fig. S5. VP1 pocket regions of CV-A10, EV-A71, CV-A16, EV-D68, CV-B3, and PV-1. **(a-f)** VP1 pocket regions of CV-A10 **(a)**, EV-A71 **(b)**, CV-A16 **(c)**, EV-D68 **(d)**, CV-B3 **(e)**, and PV-1 **(f)**. VP1 pocket regions of these six viruses are shown as transparent surface, with six pocket factors colored by elements (Oxygen, nitrogen, and carbon in red, dark blue, and magenta, respectively). The VP1 residues (colored by residues) within a distance of 4.0 Å from any atom of the corresponding pocket factor are also shown in every panel.

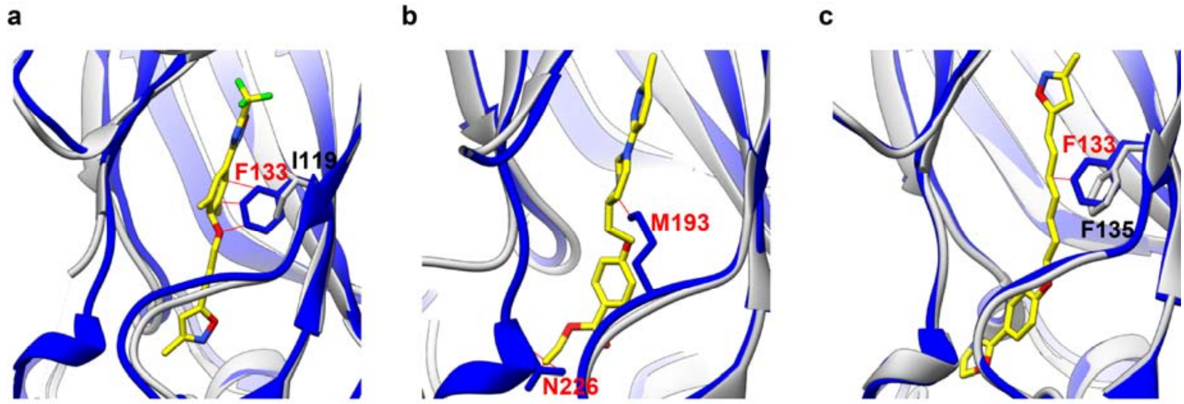

Supplementary Fig. S6. The VP1 pocket of CV-A10 is unsuitable for binding with pleconaril, pirodavir, and WIN51711. **(a)** Superposition of the VP1 pocket regions in CV-A10 (blue) and EV-D68 complexed with pleconaril (PDB: 4WM7, gray). The pleconaril is shown as sticks and colored by elements. Shown also are the residue F133 in CV-A10. Red lines indicate a distance closer than 2.4 Å between a given atom of CV-A10 (blue) and a given atom of pleconaril. The rendering style was followed throughout. **(b)** Superposition of the VP1 pocket regions in CV-A10 (blue) and poliovirus-3 complexed with pirodavir (PDB: 1VBC, gray). Shown also are the residues N226 and M193 in CV-A10. **(c)** Superposition of the VP1 pocket regions in CV-A10 (blue) and EV-A71 complexed with WIN51711 (PDB: 3ZFG, gray). Shown also are the residues F133 of CV-A10 and F135 of EV-A71, which adopt different conformations.

Supplementary Table S1. Cryo-EM data collection and refinement statistics

	CV-A10 mature virion	CV-A10 NEP
**Data collection**		
EM equipment	Titan Krios	Titan Krios
Voltage(kV)	300	300
Detector	K2 Summit	K2 Summit
Pixel size (Å)*	0.67	0.67
Electron dose (e^-/Å^2)	38	38
Exposure time (s)	7.6	7.6
Frames	38	38
Defocus range (μm)	-0.6 to -2.0	-0.6 to -2.0
**Reconstruction**		
Software	jspr	jspr
Raw micrographs	2,303	2,303
Final particles	13,273	23,312
Final resolution (Å)	2.84	3.12
Map-sharpening B factor (Å^2)	-109.4	-125.6
**Atomic modeling**		
Software	Phenix & COOT	Phenix & COOT
RMSD		
Bond length	0.009	0.010
Bond angle	1.20	1.23
Ramachandran plot (%)		
favored	94.45	92.55
allowed	5.18	7.15
outliers	0.37	0.30
Rotamer outliers (%)	0.0	0.0

*Pixel size in superresolution mode. The physical pixel size is 1.34 Å per pixel.

Supplementary Table S2. Protein-protein interactions in mature virion and NEP of CV-A10

Interface	Interface area (A^2)	
	**CV-A10 mature virion**	**CV-A10 NEP**
Interface within protomer		
VP1/VP2	2237.8	1711.5
VP1/VP3	3556.5	2876.8
VP2/VP3	1582.3	1565.4
Interface between protomers but within pentamer		
VP1/VP1	839	755
VP1/VP2	436	0
VP1/VP3	1563	1189
VP2/VP3	684	313
VP3/VP3	791	677
Interface between pentamers		
VP1/VP1	775	0
VP1/VP2	1456	6
VP2/VP2	534	108
VP2/VP3	2336	2183
VP3/VP3	79	48

Supplementary Table S3. Comparison of residues lining the VP1 hydrophobic pockets of CV-A10, EV-A71, CV-A16, EV-D68, CV-B3, and PV-1

CV-A10	EV-A71 (3VBF)	CV-A16 (5C4W)	EV-D68 (4WM8)	CV-B3 (1COV)	PV-1 (1VBD)
**Entrance and head of the pocket**					
110 Asp	112 Asp	112 Asp	96 Asn	93 Thr	111 Thr
111 Ile	113 Ile	113 Leu	97 Thr	94 Pro	112 Tyr
112 Met	114 Thr	114 Met	98 Lys	95 Arg	113 Lys
129 Phe	131 Phe	131 Phe	115 Phe	112 Phe	130 Phe
199 Tyr	201 Tyr	201 Tyr	193 Tyr	189 Tyr	205 Tyr
201 Trp	203 Trp	203 Trp	195 Val	191 Asn	207 His
223 Cys	225 Met	225 Cys	212 Asn	209 Thr	232 Ala
225 Asn	227 Asn	227 Asn	214 Ala	210 Leu	233 Ser
226 Asn	228 Asn	228 Asn	215 Asp	211 Asn	235 Asn
227 Met	229 Met	229 Met	216 Thr	212 Asn	236 Asp
273 Lys	274 Lys	274 Lys	262 Ile	259 Lys	282 Pro
**Middle part of the pocket**					
90 Val	90 Val	90 Val	69 Val	74 Val	87 Val
108 Asp	110 Asp	110 Asp	94 Thr	91 Val	109 Lys
109 Ile	111 Ile	111 Ile	95 Ile	92 Ile	110 Ile
131 Ala	133 Ala	133 Ala	117 Ala	114 Leu	132 Met
133 Phe	135 Phe	135 Phe	119 Ile	116 Leu	134 Leu
190 Val	192 Val	192 Val	184 Ile	180 Val	196 Val
193 Met	195 Met	195 Met	187 Met	183 Leu	199 Val
228 Met	230 Met	230 Met	217 Ile	213 Met	237 Phe
251 Met	253 Met	253 Met	241 Met	237 Phe	261 Leu
**End of the pocket**					
107 Trp	109 Thr	109 Trp	93 Trp	90 Trp	108 Trp
135 Phe	137 Phe	137 Phe	121 Ile	118 Phe	136 Phe
153 Tyr	155 Phe	155 Tyr	147 Phe	143 Tyr	159 Tyr
188 Val	190 Val	190 Val	182 Met	178 Met	194 Ile
231 Phe	233 Phe	233 Phe	200 Leu	216 Leu	240 Leu
249 Val	251 Ile	251 Val	239 Val	235 Ile	259 Val

Supplementary Table S4. Inhibition concentrations of small compounds against several enteroviruses

Inhibition concentration (ug/ml)	CV-A10/ S0148b	CV-A10/ Kowalik	CV-A10/ S0273b	EV-A71/ G082	CV-A16/ SZ05	EV-D68/ Fermon	EV-D68/ VR-1823
WIN 51711	>100	>100	>100	100	12.5	50	<0.78
Pleconaril	>100	>100	>100	>100	50	1.56	12.5
Pirodavir	>100	>100	>100	6.25	25	25	6.25

*The highest and lowest concentration of drug used in this experiment are 100 ug/ml and 0.78 ug/ml, respectively.

Supplementary Table S5. Docking scores of active compounds acquired by Glide 6.9 in SP mode

Virus	Compound	Docking Score (kcal/mol)
CV-A10	ICA16	-8.98
CV-A10	ICA17	-10.91
CV-A10	ICA25	-8.54
CV-A10	ICA135	-8.88
CV-A10	Pocket factor (SPH)	-7.84*
EV-A71 (3VBF)	ICA135	-8.09
EV-A71 (3VBF)	Pocket factor (SPH)	-7.53*
CV-A16 (5C4W)	ICA135	-8.00
CV-A16 (5C4W)	Pocket factor (SPH)	-7.70*
EV-D68 (4WM8)	ICA135	-10.819
EV-D68 (4WM8)	Pirodavir (4WM7)	-10.138*
CV-B3 (1COV)	PLM (1COV)	-6.612*
CV-B3 (1COV)	ICA135	-11.416
Poliovirus 1 (1VBD)	J78	-9.066*
Poliovirus 1 (1VBD)	ICA135	-9.466

* -7.84 kcal/mol was obtained by re-docking the pocket factor into the structure of CV-A10.

* -7.53 kcal/mol was obtained by re-docking the pocket factor into the structure of EV-A71 (3VBF).

* -7.70 kcal/mol was obtained by re-docking the pocket factor into the structure of CV-A16 (5C4W).

* -10.138 kcal/mol was obtained by re-docking Pirodavir into the structure of EV-D68 (4WM7).

* -6.612 kcal/mol was obtained by re-docking the pocket factor into the structure of CV-B3 (1COV).

* -9.066 kcal/mol was obtained by re-docking the pocket factor into the structure of Poliovirus 1 (1VBD).

Supplementary Table S6. Primers for qPCR analysis

Primers	Virus target	Sequence
CV-A10-RT-F	CV-A10	5’-GGGCTTTGTCCAACTCCGCA-3’
CV-A10-RT-R	CV-A10	5’-CGGACGAGCCTCTCCACTCT-3’
CV-A16-RT-F	CV-A16	5’-ATCCAGTAAGGATCCCAGACT-3’
CV-A16-RT-R	CV-A16	5’-GATTTGCATAGTGGAGAGCAG-3’
EV-A71-RT-F	EV-A71	5’-AGTTGTGCAAGGATGCTAGT-3’
EV-A71-RT-R	EV-A71	5’-CTCGTCACTAGCATTTGATG-3’
CV-B3-RT-F	CV-B3	5’-AGACCAGGCTGAATGCTA-3’
CV-B3-RT-R	CV-B3	5’-ATCCGCACTCCTCTACTG-3’
PV1-RT-F	PV1	5’-GGTTTTGTGTCAGCGTGTAATGA-3’
PV1-RT-R	PV1	5’-AAGAGGTCTCTATTCCACAT-3’
EV-D68-RT-F	EV-D68	5’-CGAGAGCATCATCAAAACAGCGACC-3’
EV-D68-RT-R	EV-D68	5’-CACTGTGCGAGTTTGTATGGCTTCT-3’
β-actin-RT-F	β-actin	5’-GGACTTCGAGCAAGAGATGG-3’
β-actin-RT-R	β-actin	5’-AGCACTGTGTTGGCGTACAG-3’
